# P-1922. Factors Influencing 90-Day Mortality in Non-HIV, Non-Transplant Patients with Cryptococcal Meningoencephalitis

**DOI:** 10.1093/ofid/ofaf695.2091

**Published:** 2026-01-11

**Authors:** Ameera Jamshad, Bayard A Taylor, Daniel B Chastain, Charlie A Garcia, Andrés F Henao Martínez

**Affiliations:** The University of Georgia College of Pharmacy, Marietta, GA; University of Georgia, Ellerslie, GA; University of Georgia College of Pharmacy, Albany, GA; Colorado School of Public Health Anschutz, Aurora, Colorado; University of Colorado Anschutz Medical Campus, Aurora, Colorado

## Abstract

**Background:**

Cryptococcal meningoencephalitis (CM) is increasingly recognized in individuals without HIV or a history of solid organ transplantation (NHNT). Emerging US data suggest higher CM-related mortality among NHNT individuals compared to those with HIV. This study aimed to characterize NHNT patients with CM and identify differences between survivors and non-survivors.

**Figure:**
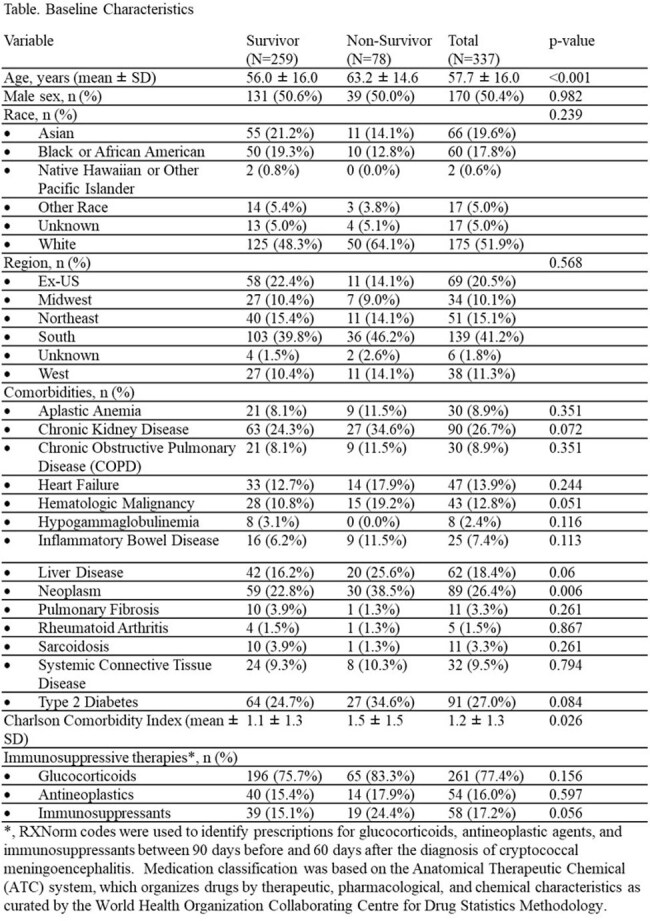

**Methods:**

We identified adult patients (≥18 years) diagnosed with CM between 2003 and 2024 using data from TriNetX, a global federated health research network. CM diagnosis was defined by an ICD-10-CM code for cerebral cryptococcosis or laboratory evidence of cryptococcal antigen or DNA in cerebrospinal fluid. Inclusion was limited to patients treated with amphotericin B to increase the specificity of CM diagnosis. Individuals with HIV and a history of solid organ transplantation were excluded. Demographics, comorbidities, use of immunosuppressive therapies, and 90-day mortality were described, with comparisons made between survivors and non-survivors.

**Results:**

Among 337 NHNT patients with CM, the mean age was 58 years, 50% were male, and 52% identified as White (Table). Most patients resided in the South (41%), with 21% located outside the US. Common comorbidities included type 2 diabetes (27%), chronic kidney disease (27%), and neoplasms (26%). The overall 90-day mortality rate was 23%. Compared with survivors, non-survivors were older (63 vs 56 years, p< 0.001) and had a higher Charlson Comorbidity Index (1.5 vs 1.1, p=0.026). Non-survivors also had a higher prevalence of neoplasms (39% vs 23%, p=0.006). Glucocorticoid use was high in both groups (83% vs 76%) but did not differ significantly, nor did the use of antineoplastic or other immunosuppressive agents.

**Conclusion:**

NHNT patients with CM experienced a high 90-day mortality rate of 23%, with older age, greater comorbidity burden, and neoplastic disease associated with non-survival. Immunosuppressive therapy use was common but did not differentiate outcomes, suggesting that underlying host factors, such as immune dysregulation or the direct impact of comorbidities, may be more influential in determining clinical outcomes.

**Disclosures:**

Andrés F. Henao Martínez, MD, MPH, F2: Grant/Research Support|Scynexis: Grant/Research Support

